# *QuickStats:* Percentage Distribution* of Long-Term Care Staffing^†^ Hours,^§^ by Staff Member Type and Sector — United States, 2016

**DOI:** 10.15585/mmwr.mm6717a6

**Published:** 2018-05-04

**Figure:**
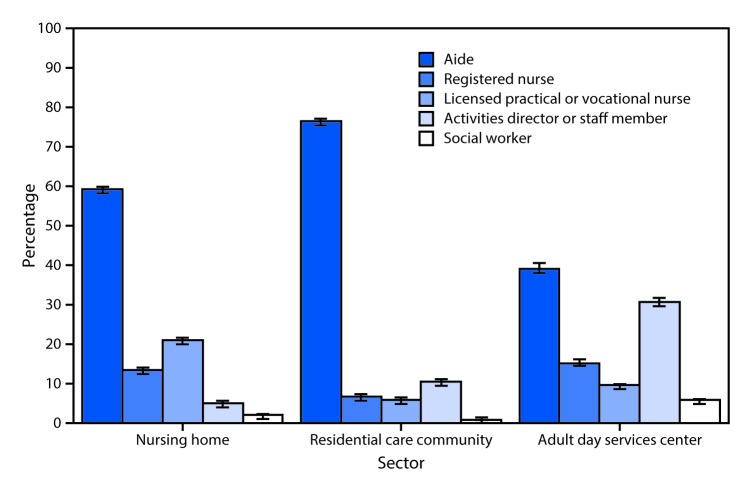
In 2016, aides provided more hours of care in the major sectors of long-term care than the other staffing types shown. Aides accounted for 59% of all staffing hours in nursing homes, compared with licensed practical or vocational nurses (21%), registered nurses (13%), activities staff members (5%), and social workers (2%). Aides accounted for 76% of all staffing hours in residential care communities, in contrast to activities staff members (10%), registered nurses (7%), licensed practical or vocational nurses (6%), and social workers (1%). In adult day services centers, aides provided 39% of all staffing hours, followed by activities staff members (30%), registered nurses (15%), licensed practical or vocational nurses (9%), and social workers (6%).

**Source:** National Study of Long-Term Care Providers, 2016. https://www.cdc.gov/nchs/nsltcp/index.htm.

